# Spatial characteristics of nutrient allocation for *Picea crassifolia* in soil and plants on the eastern margin of the Qinghai-Tibet Plateau

**DOI:** 10.1186/s12870-023-04214-x

**Published:** 2023-04-17

**Authors:** Jingjing Wu, Liang Jiao, Huijun Qin, Xichen Che, Xuli Zhu

**Affiliations:** 1 College of Geography and Environmental Science, Northwest Normal University, No. 967, Anning East Road, Lanzhou, 730070 China; 2 Key Laboratory of Resource Environment and Sustainable Development of Oasis, Gansu Province, Northwest Normal University, Lanzhou, 730070 China

**Keywords:** Stoichiometric characteristics, Coupling relationship, Spatial characteristics, Qinghai spruce, Qinghai-Tibet Plateau

## Abstract

**Background:**

Understanding the stoichiometric characteristics and nutrient allocation strategies of dominant tree species in montane forest systems can provide a basis for decision-making in relation to montane system management. Therefore, according to precipitation and temperature gradients, we selected three typical areas in the Qilian Mountains on the eastern margin of the Qinghai-Tibet Plateau to analyse the spatial relations of plant-soil stoichiometric characteristics and nutrient allocation strategies of plant tissues for Qinghai spruce (*Picea crassifolia*) along different environmental gradients.

**Results:**

1) The plant and soil stoichiometric characteristics had similar spatial patterns. The C content of plants and soils tended to decrease with increasing latitude, and the N and P contents and the N:P ratio tended to increase with increasing latitude. 2) The stoichiometric characteristics of the plant tissues also interacted with each other and showed synergistic trade-offs. Nutrient allocation in the eastern section of the Qilian Mountains was similar to that in the western section, while more N and P in the plant stems were allocated to maintain plant growth in the relatively arid western Sect. 3) The nutrient allocation strategies in the plant tissues were mainly regulated by soil and climate.

**Conclusions:**

Information on plant-soil stoichiometric characteristics along different gradients can help us better understand the nutrient patterns and dynamics of forest ecosystems under arid and semiarid conditions at a wide geographic scale from the perspective of plant nutrient partitioning.

**Supplementary Information:**

The online version contains supplementary material available at 10.1186/s12870-023-04214-x.

## Introduction

Plant-soil material cycles and energy flows have an important impact on terrestrial ecosystems [1], and the spatial coupling between plants and soil can be used to understand the geochemical cycles and ecological functions of terrestrial ecosystems [2]. The crucial roles of plant-soil stoichiometry in indicating the soil interior nutrient cycling and plant nutrient supply of forest ecosystems have been widely verified, whereas this information has been less explored when considering the influencing factors regionally, especially plants along particular environmental gradients. Therefore, exploring the spatial coupling of plant-soil carbon (C), nitrogen (N), and phosphorus (P) stoichiometric characteristics across environmental gradients can help us better understand nutrient patterns in terrestrial ecosystems and their potential impact on ecosystem processes under environmental change [3, 4].

Plant-soil stoichiometric characteristics have different spatial patterns along different environmental gradients. Plant-soil C, N, and P concentrations increase significantly in desert grassland ecosystems with increasing latitude, while C and N do not change significantly in alpine grassland ecosystems [5]. On the Loess Plateau, the leaf N:P ratios increase with latitude [6]. In contrast, globally, with decreasing MAT, the leaf N and P contents in wetland plants increase, while the leaf N:P ratio decreases [4, 7, 8]. In addition, research has found a gradual increase in N and P in plant leaves from the tropics to cooler and drier regions [9]. This spatial variation in plant-soil stoichiometric characteristics across environmental gradients is known as convergent plant adaptation [10]. Analyses of the differences in plant-soil stoichiometric characteristics along different environmental gradients can provide us with a better understanding of nutrient cycling and energy flow processes between plants and soils in terrestrial ecosystems.

The allocation of limited plant nutrients, as an important strategy for plant responses to environmental changes, reflects the influence of plant evolutionary and ecological processes and the trade-offs of multiple functions [11, 12]. To maximize plant growth and maintain optimal metabolic activities, plants need to balance nutrient allocation among organs under different stresses [13]. For example, the nutrient allocation strategy of herbaceous plants is by regulating N:P ratios among branches, stems, and leaves. Legumes allocate more N and P to stems and branches, but deciduous broadleaf plants allocate more nutrients to leaves [14]. In arid ecosystems, plants will increase leaf nutrient concentrations to increase the potential photosynthetic capacity per unit area of leaf for high water use efficiency [15]. In addition, plants under drought conditions allocate N to the leaves to compensate for the low photosynthetic rate caused by reduced stomatal conductance [16]. The growth of plants in arid desert areas is vulnerable to water stress. Increasing the N content of leaves makes it possible for plants to adapt to arid environments [17]. There are significant differences in the contents of chemical elements among various organs of *Ammopiptanthus mongolicus. *Leaves usually have higher nutrient contents than stems and roots to maintain their higsh physiological and ecological activities [18, 19]. The trade-off between the chemometric characteristics of plant tissues reflects the regulatory strategies for plants to acquire resources and allocate nutrients in different habitats. Studying trade-offs between regional plant organ chemistry characteristics can help us better understand plant growth strategies at the regional scale.

Currently, many scholars have focused on the coupling relationship between plant tissue chemometric characteristics and environmental elements. Specifically, it has been suggested that over 90% of plant N and P is derived from nutrients returned to the soil by the plant in the previous year [20]. Soil nutrient content is an important factor in regulating plant C:N:P stoichiometry, and plant nutrient acquisition strategies vary with soil age [21]. Plant stoichiometric characteristics are influenced by soil nutrient content, and climate also plays a role in their spatial patterns. Several hypotheses have been proposed, including the stoichiometric endostability theory [22], the limiting element stability hypothesis [23], the leaf nutrient content stability hypothesis [24], the temperature-plant physiology hypothesis, and the temperature-biogeochemistry hypothesis [4, 25]. The temperature-plant physiology hypothesis refers to leaves having higher N and P in cooler climates to compensate for reduced biochemical reaction efficiency and rates. In contrast, the temperature-biogeochemical hypothesis states that colder temperatures inhibit the mineralization and decomposition of organic matter, which in turn inhibits plant uptake of N and P [4]. Soil N and P concentrations both significantly decrease with increasing mean annual temperature and mean annual precipitation [26]. However, on the Loess Plateau, the leaf N:P ratio increases as the latitude and annual solar radiation increase and the mean annual rainfall and mean annual temperature decrease [6]. These results suggest that plant stoichiometric drivers are regionally specific rather than universally applicable. Therefore, we selected three typical regions in the Qilian Mountains region at the eastern margin of the Qinghai-Tibet Plateau to further determine the biogeographic patterns of plant tissues and soil nutrients at the regional scale and the nutrient allocation strategies of plant tissues along different environmental gradients.

The Qilian Mountains are located on the eastern margin of the Tibetan Plateau and are an important ecological barrier that is vulnerable to human disturbance [27]. The unique climatic characteristics and complex topographic features lead to significant differences in temperature and precipitation along the latitudinal gradient, thus providing an ideal natural laboratory for studying changes in plant tissue nutrient characteristics in response to soil and climate [28, 29]. As a dominant plant species in the Qilian Mountains, Qinghai spruce (*Picea crassifolia*) has important ecological functions. The study of its stoichiometric characteristics and soil environment can provide a more comprehensive understanding of the plant-soil nutrient dynamic balance and the nutrient trade-off distribution strategy of plant tissue nutrients among different regions. In this study, we investigated the characteristics of surface soils (0–40 cm) in the Qilian Mountains (soil organic carbon, soil total nitrogen, soil total phosphorus, their ratios, soil bulk density, and soil pH) and the characteristics of tissue traits of Qinghai spruce (C, N, P and their ratios). Thus, we aimed to answer the following questions: 1) How do soil characteristics and plant tissue (stem, leaf, branch, fine roots and thick roots) characteristics of Qinghai spruce change with geographical and climatic factors? 2) What are the nutrient partitioning trade-offs among plant tissues? 3) What are the spatial relations between soil and plant tissue stoichiometric characteristics?

## Materials and methods

### Study area

**Fig. 1:**
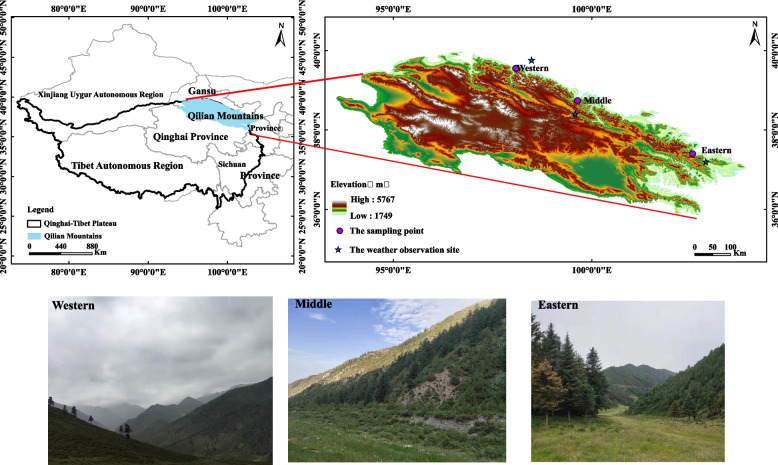
Overview map of the study areas

The Qilian Mountains are located at 93.4°E-103.4°E, 35.8°N-40.0°N (Fig. 1), and are affected by the interaction with the East Asian monsoon [30]. The topography of Qilian Mountains is high in the west and low in the east, and the mountain range is oriented in a northwest-southeast direction with an average elevation of 4000–5000 m. The Qilian Mountains have a typical continental alpine and semi-humid mountain climate with cold and dry winters and cool and humid summers, and the mean annual temperature below 4 °C, 1744 h of sunshine, and annual precipitation of about 400 mm mainly from May to September [31]. The Qilian Mountains have obvious vertical zonality, and the main vegetation includes Qinghai spruce, Qilian juniper (*Sabina przewalskii*), and Chinese pine (*Pinus tabuliformis*). As the dominant tree species in this region, Qinghai spruce is widely distributed on shady and semi-shady slopes from 2500 to 3400 m above sea level [32, 33]. In this paper, three sampling points were selected according to temperature and precipitation gradient (Table 1). We selected the weather observation site closest to the sampling point (Table S1), and the mean annual temperature and mean annual precipitation are shown in Fig. S1.

**Table 1:** The information of sampling point information

sampling point	Longitude (°)	Latitude(°)	Elevation(m)
Eastern	102.54°E	37.4°N	3200
Middle	99.64°E	38.74°N	3025
Western	98.08°E	39.55°N	3070

### Soil and plant sampling and analyses

The plant samples were collected from wild natural populations with permission from the Administration of Qilian Mountain National Nature Reserve, Gansu Province. The collection of plants and soil materials was conducted in accordance with the guidelines and legislation of the Administration of Qilian Mountain National Nature Reserve, Gansu Province. None of the species included in the present study are listed in the Convention on International Trade in Endangered Species of Wild Fauna and Flora (CITES). Formal field identification was carried out by Qiangen Fang (Gansu Agricultural University), and the specimen information of the species involved in this study is available in the Herbarium of the College of Life Sciences, Northwest Normal University. Based on the distribution characteristics of Qinghai spruce in the Qilian Mountains, three 10 m × 10 m sample squares were randomly set at each of the three sampling sites in mid-July 2021. To minimize the effects of the differences in light and needle surface temperature on elements in the trees, each sampling was conducted at noon [34]. At each sample site, five Qinghai spruce trees were selected that were upright, healthy, undamaged, nonisolated, and the same size (age, height, and diameter at breast height), and samples of leaves, stems, thick roots (> 5 mm in diameter) and fine roots (< 2 mm in diameter) were collected. Leaf and branch samples were taken from unshaded mature branches that were two years old on the uphill face. Trunk samples were obtained by drilling cores with growth cones at a 1.3 m diameter at breast height, and five cores were used as stem samples. Fine root samples were obtained via excavation at a depth of 0–40 cm into the soil layer. Three cores were drilled at the base of each sample tree, that is, the thick root samples with the highest branching grade, using growth cones. Soil samples were collected from 0–10 cm, 10–20 cm and 20–40 cm at a distance of 2 m from the trunk of each target tree. Soil samples were collected in 3 directions from the target trees, and the samples were mixed [35]. Part of the collected soil samples were used to measure soil bulk density and part was used to measure soil chemistry. The soil samples used to measure soil bulk density were dried in an oven at 75 °C for 48 h after recording the fresh weight of the soil material, and then the dry weight of the soil samples was recorded.

The collected plant litters were oven-dried at 65 ℃ until the mass was constant, Afterward, the samples ground through a 0.15 mm sieve using a mixed ball mill (MM400, Retsch, Germany), and sealed for low temperature storage [36]. Soil samples used to measure soil chemistry were naturally air-dried in the laboratory. They were handpicked to remove plant and detritus, and the samples were sieved completely through a 0.15 mm sieve and sealed for low temperature storage for soil physicochemical analysis. The organic C of Qinghai spruce and soil organic C (SOC (C)) content was analyzed by the potassium dichromate-sulfuric acid oxidation method. The N and P contents of the soil and plant samples were determined following heating digestion method by fully automated chemical analyzer (Smartchem 200, Advanced Monolithic Systems, Graz, Italy). The soil pH was determined in a soil/water (1:2.5; w/v) suspension with a pH meter. Soil bulk density (SBD) was determined by the soil core method, and the ratio of soil mass to total volume was calculated after drying to constant weight in an oven at 105 °C [37].

### Statistical analyses

One-way ANOVA was used to study the effects of three sampling points on growth characteristics of understory plants, soil physical and chemical properties, water use efficiency and plant nutrients. One-way ANOVA analysis was mainly implemented using SPSS 22.0 (SPSS Inc., Chicago, IL, USA). Least squares difference (LSD) post hoc tests were conducted to identify significant differences between means. Pearson coefficient analysis was used to examine the relationship among plant tissue traits and between plant tissue traits and soil characteristics. Based on the correlation coefficients and their significance, the trade-off synergistic relationship between plant tissues was quantified (Table S2). The plant tissue traits and soil nutrient contents were expressed as g/kg on a dry mass basis, and all of the C:N, C:P, and N:P ratios in the soil and plant tissue were measured on a mass ratio basis.

We used redundancy analysis (RDA) to quantify the specific effects of soil factors (pH, SBD, SOC, TN, TP and their ratios) and climate (MAT and MAP) on Qinghai spruce tissue traits (C, N, P and their ratios). The reason was that RDA can separate the total variance explained by multiple factors into the independent explanatory rates of each factor and the common explanatory rates of different factors [38]. RDA was conducted using R 3.4.0 software (R Development Core Team, 2017) with the “vegan” package. Significance was determined at the 0.05 level.

## Results

### Spatial pattern of soil characteristics

#### Spatial pattern of soil physical characteristics

**Fig. 2:**
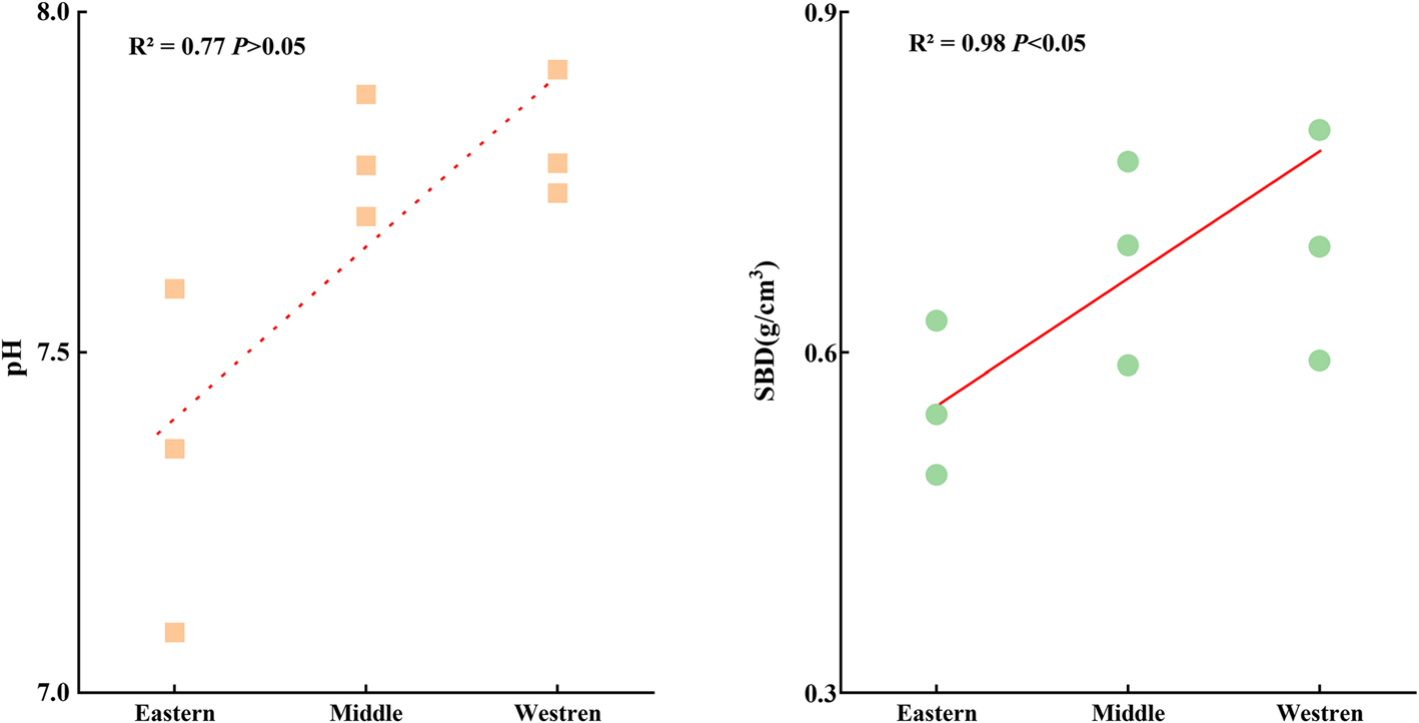
Variation of soil physical characteristics factors with latitude in the Qilian Mountains. (Note: SBD = Soil bulk density)

**Fig. 3:**
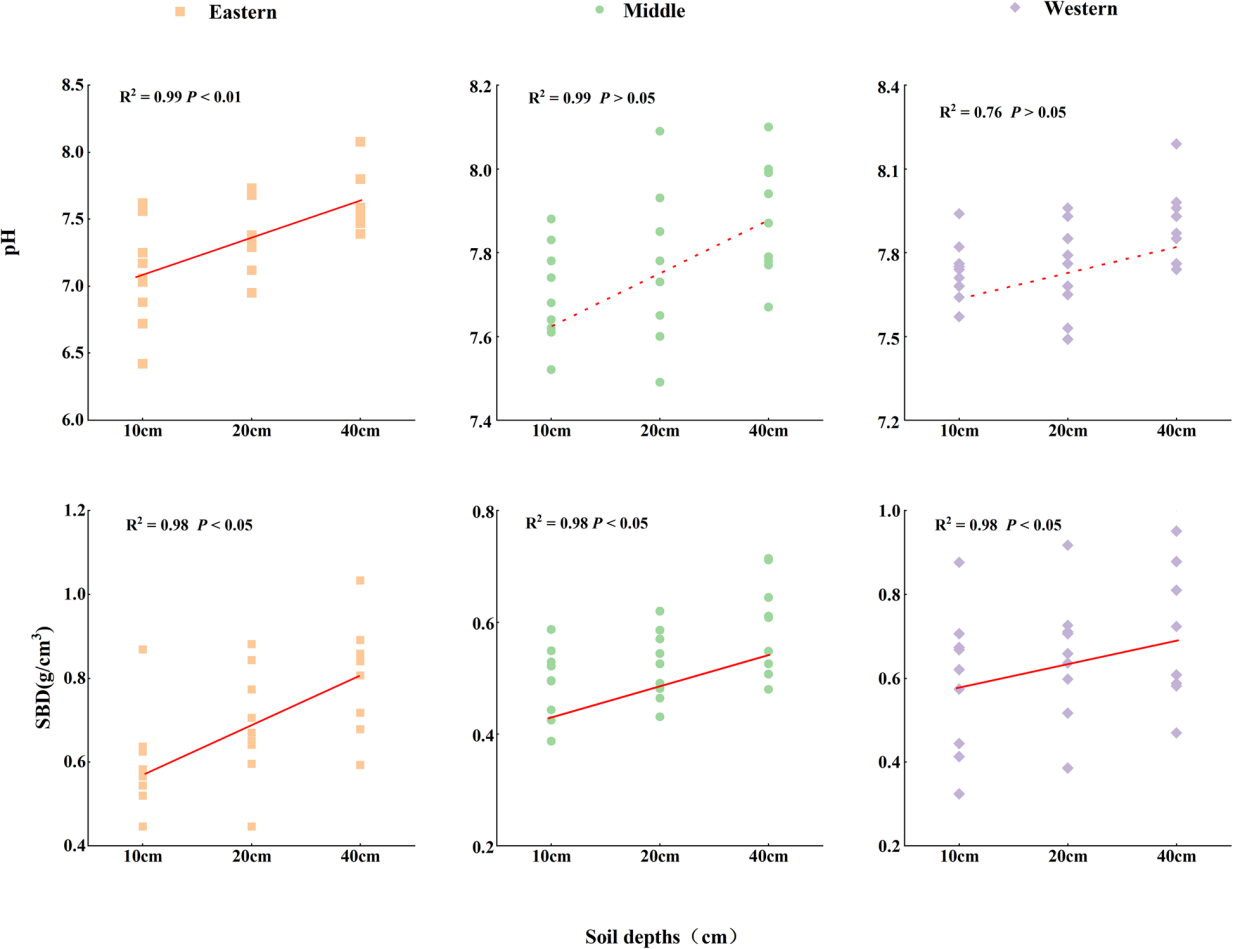
Variation of soil physical characteristics factors with soil depth in the Qilian Mountains. (Note: SBD = Soil bulk density)

Considering all of the plots, the overall mean soil pH and SBD were 7.64 ± 0.06 (range 6.42–8.19) and 0.65 ± 0.02 g/cm^3^ (range 0.08–0.99), respectively (Table 2). The physical characteristics of soil in the three regions had strong spatial heterogeneity characteristics. Horizontally, SBD showed an increasing trend from the eastern to western sections (*P* < 0.05) (Fig. 2). Vertically, the pH and SBD of the three regions showed a trend of 40 cm > 20 cm > 10 cm. The soil in the eastern Qilian Mountains was more acidic, while in the middle and western sections, the soil was mostly neutral and alkaline (Fig. 3). The SBD of 0.70 g/cm^3^ in the western section of the Qilian Mountains was higher than that of 0.68 g/cm^3^ in the middle section and 0.55 g/cm^3^ in the eastern section (Fig. 2).

**Table 2:** Statistical characteristics of physical characteristics (pH, SBD) of the overall soil layer (0–40 cm) for soil samples in the Qilian Mountains

Parameters	Min	Max	Mean ± SD	Skewness	Kurtosis	CV
pH	6.42	8.19	7.64 ± 0.06	-1.35	2.52	0.03
SBD(g/cm^3^)	0.08	0.99	0.65 ± 0.02	0.74	-0.07	0.26

the skewness and kurtosis were used to test the normality of the data. skewness = 0, indicating normal distribution, skewness > 0, indicating the right skewness distribution, skewness < 0, indicating a left-skewed distribution; Kurtosis = 3, indicating normal distribution, Kurtosis > 3, indicating fat tails, Kurtosis < 3, indicating thin tails; CV(coefficient of variation) = SD/Mean

#### Spatial patterns of soil stoichiometric characteristics

**Fig. 4:**
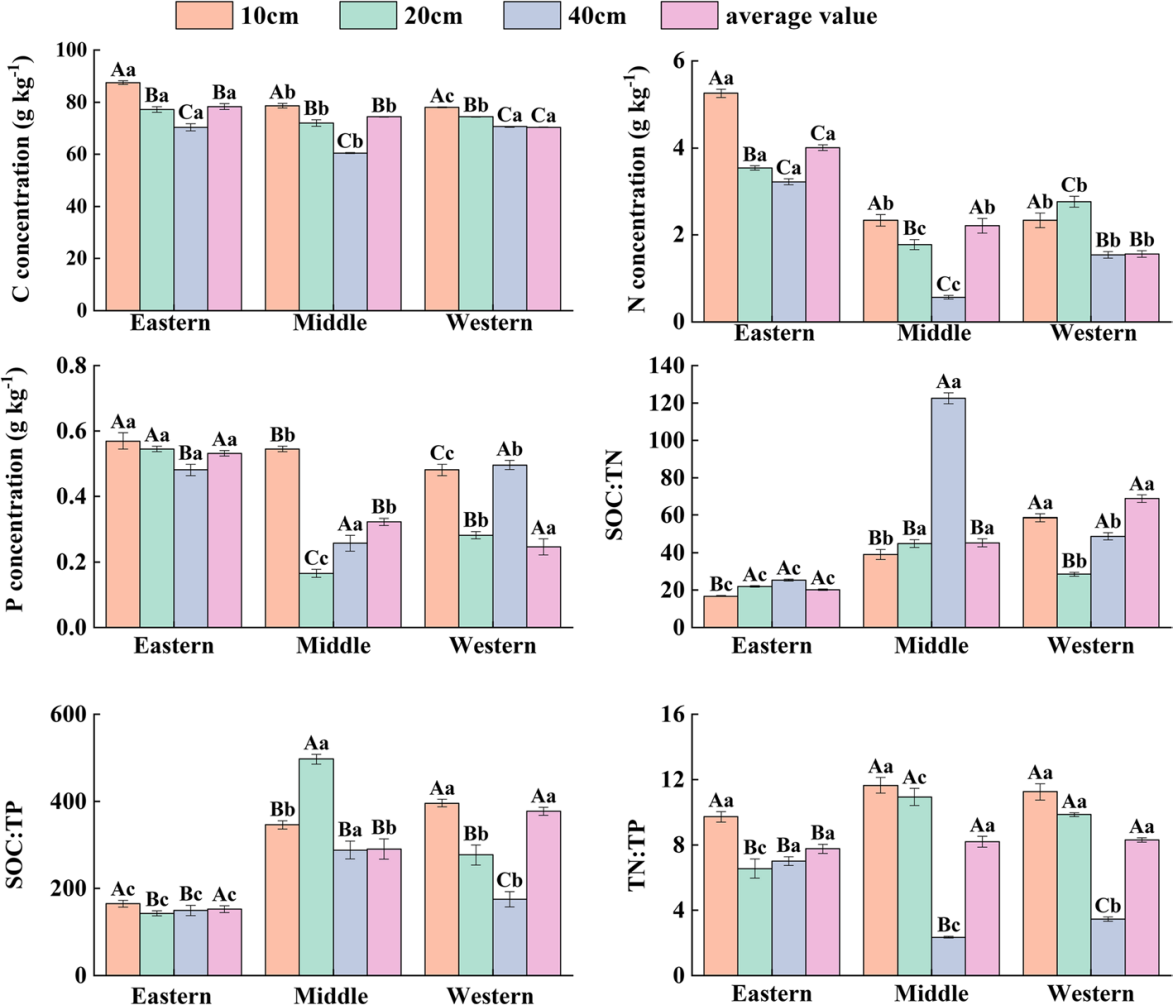
Soil stoichiometric characteristics with soil depths in the Qilian Mountains. (Note: SOC = soil organic carbon, TN = soil total nitrogen, TP = soil total phosphorus, different capital letters indicate significant differences in stoichiometric characteristics of different soil depths in the same region, and different lowercase letters indicate significant differences in stoichiometric characteristics of the same soil depth in different regions.)

Considering all of the plots, the overall mean soil SOC, TN and TP concentrations were 74.32 ± 1.99 g/kg (range 45.22–99.49), 2.59 ± 0.28 g/kg (range 0.25–5.60) and 0.37 ± 0.03 g/kg (range 0.09–0.83), respectively (Table 3). The SOC:TN, SOC:TP and TN:TP ratios of the average soil level were 44.79 ± 7.21, 273.25 ± 29.50 and 8.08 ± 0.09, respectively (Table 3). The soil stoichiometric characteristics of the three regions exhibited strong spatial heterogeneity characteristics. Horizontally, soil SOC, TN, and TP showed a decreasing trend from eastern to western, while SOC:TN, SOC:TP, and TN:TP of soil showed an increasing trend (*P* < 0.05) (Fig. 4). Vertically, the SOC concentration in the three regions decreased with increasing soil depth. The N and P concentrations of soil in the eastern section decreased with increasing soil depth (*P* < 0.05), while those in the western section decreased and then increased (Fig. 4).

**Table 3:** Statistical characteristics of the overall soil layer (0–40 cm) nutrient traits (SOC, TN, TP and SOC:TN:TP ratio) for soil samples in the Qilian Mountains

Parameters	Min	Max	Mean ± SD	Skewness	Kurtosis	CV
SOC(g/kg)	45.22	99.49	74.32 ± 1.99	-0.51	2.12	0.14
TN(g/kg)	0.25	5.06	2.59 ± 0.28	0.37	-0.55	0.58
TP(g/kg)	0.09	0.83	0.37 ± 0.03	0.55	-0.69	0.56
SOC:TN	14.19	177.35	44.79 ± 7.21	2.18	5.28	0.84
SOC:TP	88.08	705.96	273.25 ± 29.50	0.99	0.64	0.56
TN:TP	1.99	17.64	8.08 ± 0.09	0.70	0.15	0.58

the skewness and kurtosis were used to test the normality of the data. skewness = 0, indicating normal distribution, skewness > 0, indicating the right skewness distribution, skewness < 0, indicating a left-skewed distribution; Kurtosis = 3, indicating normal distribution, Kurtosis > 3, indicating fat tails, Kurtosis < 3, indicating thin tails; CV(coefficient of variation) = SD/Mean

### Spatial patterns of plant stoichiometric characteristics

#### Spatial pattern variation of plant C, N and P contents and their ratios

**Fig. 5:**
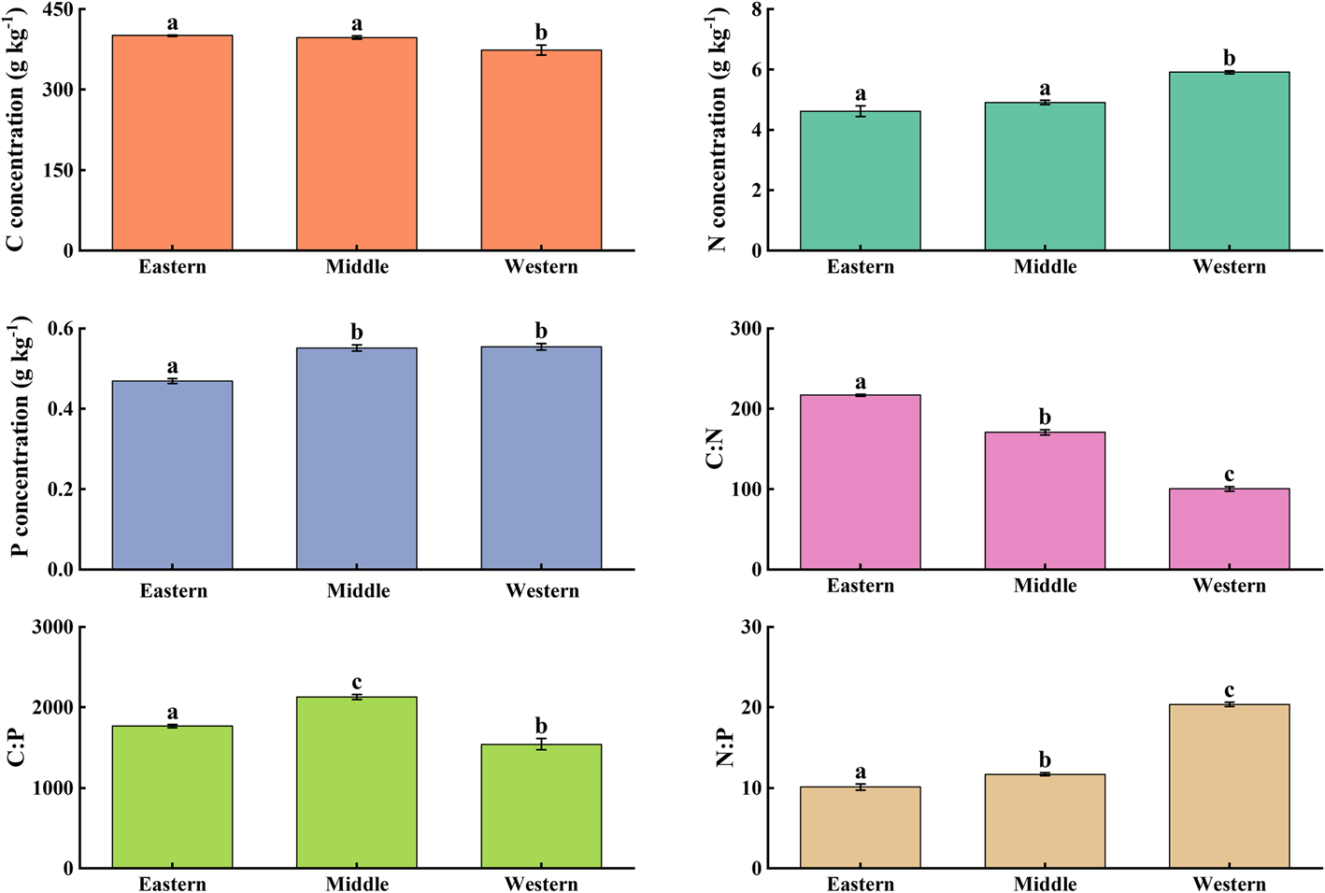
Variation of plant stoichiometry characteristics with latitudes in the Qilian Mountains (Different lowercase letters indicate significant differences in stoichiometric characteristics in different regions)

**Fig. 6:**
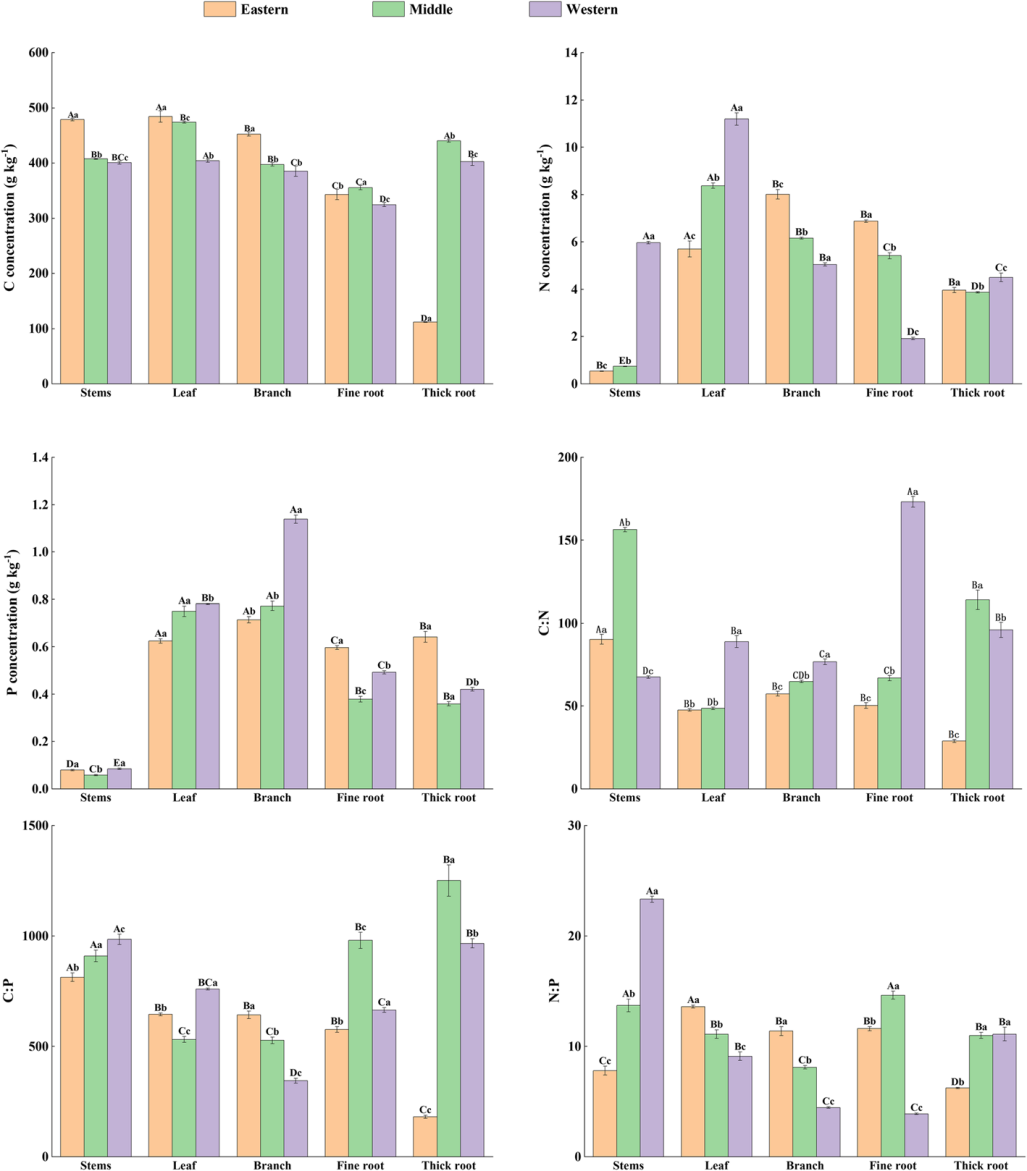
Stoichiometric characteristics of various tissues of plants in the Qilian Mountain. (Different capital letters indicate significant differences in stoichiometric characteristics of different plant tissues in the same region, and different lowercase letters indicate significant differences between the stoichiometric characteristics of the same plant tissue in different regions.)

Considering all of the plots, the overall mean plant C, N and P concentration were 391.49 ± 3.16 g/kg (range 45.22–99.49), 5.13 ± 0.05 g/kg (range 0.25–5.60) and 0.52 ± 0.01 g/kg (range 0.09–0.83), respectively (Table 4). The C:N, C:P and N:P ratios average levels were 163.31 ± 3.33, 1838.92 ± 20.72 and 13.88 ± 0.18, respectively (Table 4). The stoichiometric characteristics of the three regions showed spatial trend characteristics. In the horizontal space, the C concentration of plant showed that the eastern Sect. (400.88 g/kg) > the Middle Sect. (397.94 g/kg) > the western Sect. (373.92 g/kg), while N, P, and N:P in the plant increased with increasing latitude (*P* < 0.05) (Fig. 5). There was spatial heterogeneity in plant tissue stoichiometric characteristics (Table 5). The C concentration of plant stems, leaves, and branches decreased, the N concentration of plant branches and fine roots decreased, the N concentration of leaves and stems increased, the P concentration of plant leaves and branches increased, the C:N of plant leaves, branches, and fine roots increased from eastern to western. Meanwhile, the C:P of plant branch showed that eastern Sect. (641.59) > middle Sect. (526.91) > western Sect. (343.35), while stem C:P was western Sect. (984.67) > middle Sect. (908.76) > eastern Sect. (812.65). The N:P of stems and thick roots increased with latitude, and the opposite was true for leaf and branch N:P (Fig. 6).

**Table 4:** Statistical characteristics of the stoichiometric characteristics (C, N, P and C:N:P ratio) of plant samples from Qilian Mountains

Parameters	Min	Max	Mean	Skewness	Kurtosis
C (g/kg)	108.50	703.51	391.49 ± 3.16	-0.82	3.73
N(g/kg)	0.49	11.16	5.13 ± 0.05	-0.04	-0.52
P(g/kg)	0.03	1.30	0.52 ± 0.01	0.10	-0.37
C:N	23.31	1005.19	163.31 ± 3.33	2.45	5.10
C:P	133.43	9616.55	1838.92 ± 20.72	1.87	2.29
N:P	3.13	99.45	13.88 ± 0.18	3.81	15.20

**Table 5:** Statistical characteristics of nutrient traits (C, N, P and C:N:P ratio) of individual tissues (stems, leaves, branches, fine roots, and thick roots) of plant samples in the Qilian Mountains

	Stems	Leaf	Branch	Fine root	Thick root
C (g kg^−1^)	427.21 ± 10.51	450.26 ± 14.04	410.44 ± 12.87	342.10 ± 10.08	327.48 ± 8.49
N (g kg^−1^)	2.28 ± 0.71	8.1 ± 0.59	6.38 ± 0.38	4.78 ± 0.59	4.08 ± 0.21
P (g kg^−1^)	0.07 ± 0.005	0.72 ± 0.03	0.86 ± 0.05	0.48 ± 0.03	0.46 ± 0.04
C:N	104.62 ± 6.23	60.64 ± 6.48	66.12 ± 3.09	94.39 ± 5.75	82.18 ± 2.89
C:P	902.23 ± 60.21	636.01 ± 31.72	505.72 ± 41.72	758.14 ± 65.70	833.56 ± 51.07
N:P	26.22 ± 5.71	11.24 ± 0.76	7.98 ± 0.72	10.38 ± 1.36	9.55 ± 0.91

### Patterns of nutrient allocation between plant tissues

**Fig. 7:**
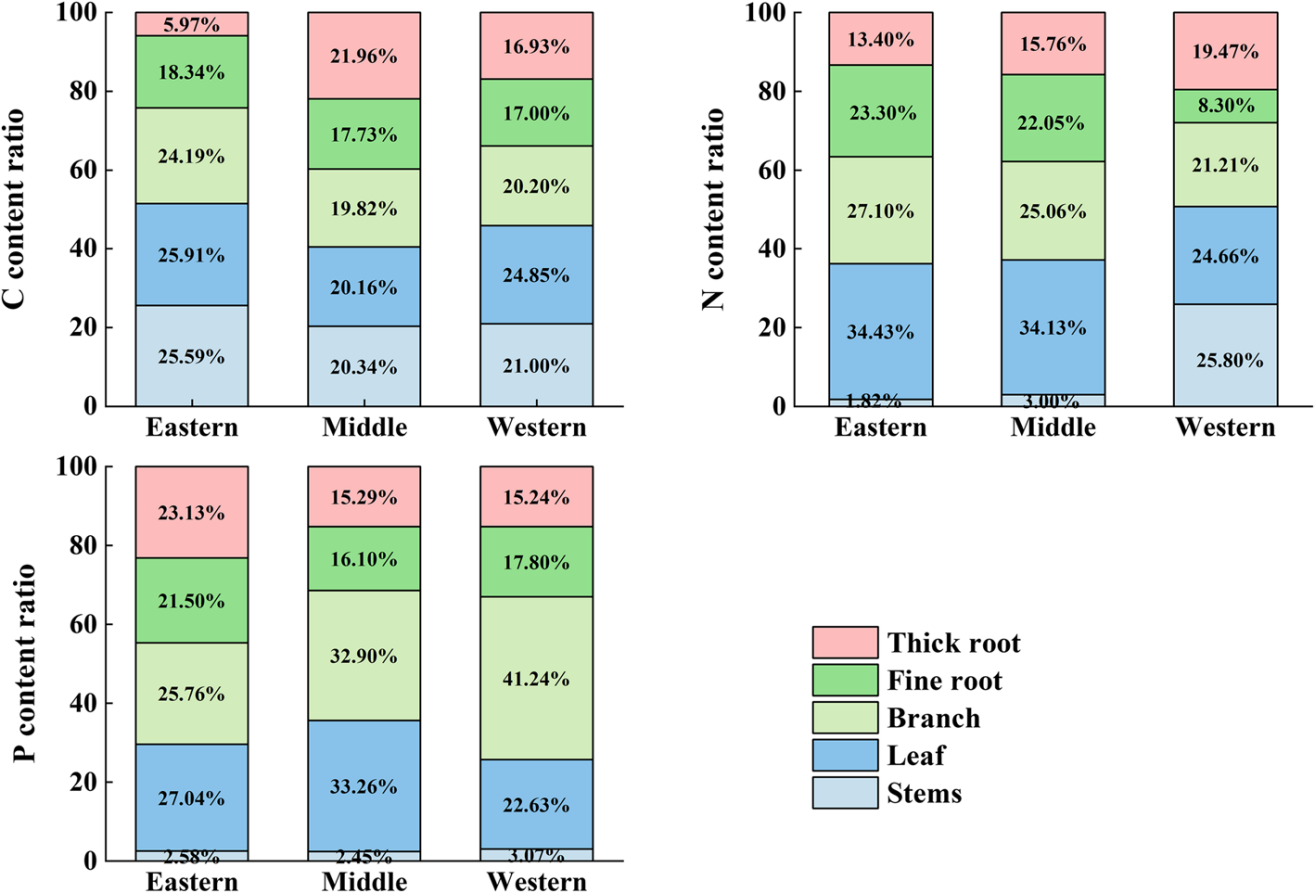
stoichiometry characteristics of plants as a percentage of each tissue in the Qilian Mountains

There were differences in nutrient distribution trade-offs among plant tissues in the same region. Figure 7 shows that there were differences in the distribution of C, N, and P contents among plant tissues in the three regions. Plant tissues in the eastern section of the Qilian Mountains allocated less C to the thick roots, while the tissues in the middle and western sections were more evenly distributed. In addition, the plant tissues in the eastern and middle sections of the Qilian Mountains allocated less N to the stems, while tissues in the relatively arid western section of the Qilian Mountains allocated more N to the stems. The pattern of plant allocation to P content was relatively similar in the three regions, with less allocation to plant stems and more allocation to plant branches.

### Relationship of plant nutrient contents with soil characteristics and climate

#### Effects of soil and climate on plant stoichiometric characteristics

**Fig. 8:**
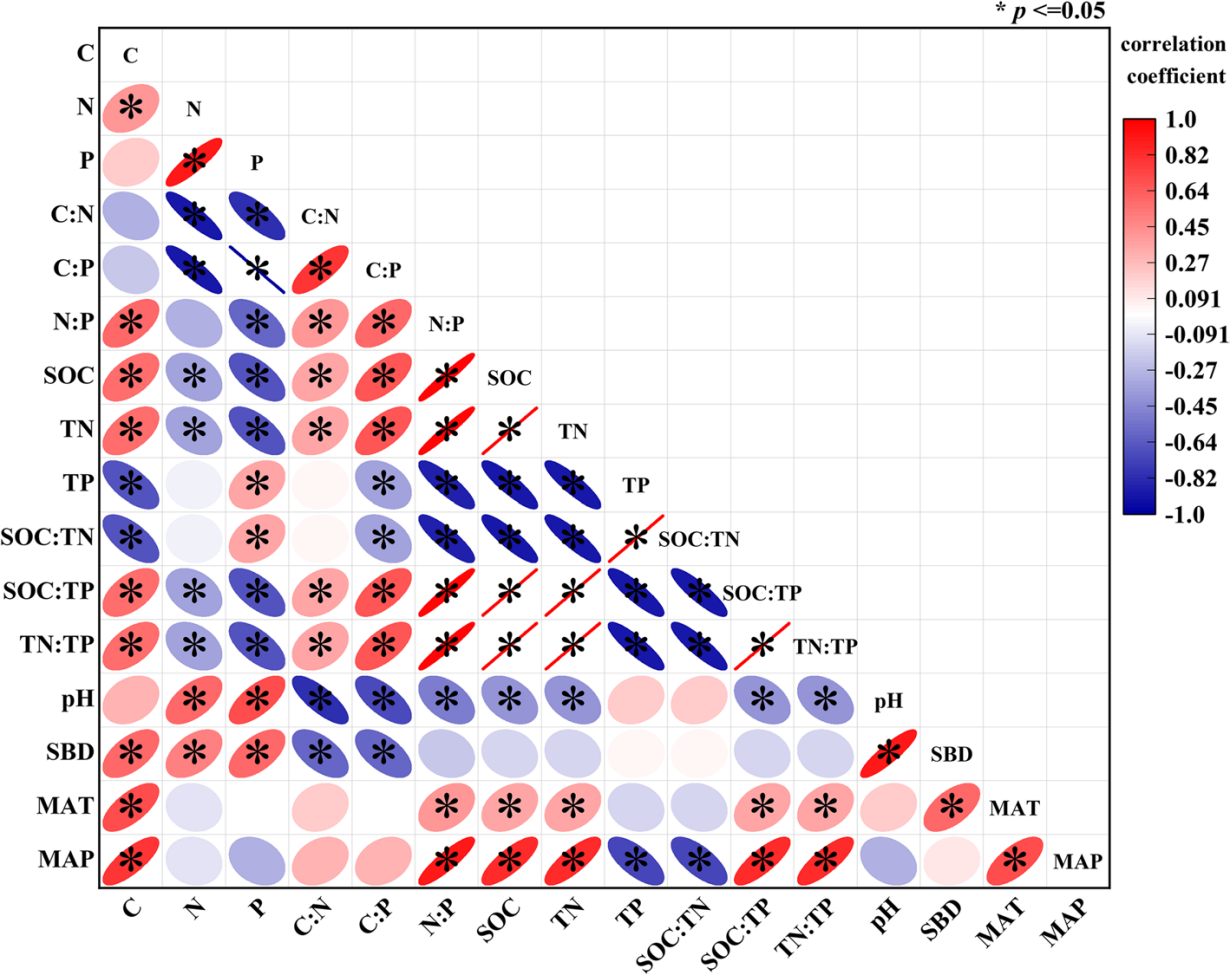
Relationships between plant stoichiometry characteristics and soil, climate in the Qilian Mountains. (Note: The width of the ellipse indicates the degree of influence, blue indicates negative correlation, red indicates positive correlation, C = carbon concentration of Qinghai spruce, N = nitrogen concentration of Qinghai spruce, P = phosphorus concentration of Qinghai spruce, SOC = soil organic carbon, TN = soil total nitrogen, TP = soil total phosphorus, pH = soil pH, SBD = soil bulk density, MAT = mean annual temperature, MAP = mean annual precipitation)

Plant C, N and P concentrations and their ratios were influenced by soil and climate. The plant C concentration was positively correlated with SOC, TN, SBD, MAT, and MAP and negatively correlated with TP and SOC:TN (*P* < 0.05). Plant N content was positively correlated with pH and SBD and negatively correlated with SOC, TN, SOC:TP, and TN:TP (*P* < 0.05). The plant P concentration was positively correlated with TP, SOC:TN, pH, and SBD and negatively correlated with SOC, TN, SOC:TP, and TN:TP (*P* < 0.05). The C:N and C:P of plants were both positively correlated with SOC, TN, SOC:TP, and TN:TP and negatively correlated with pH and SBD (*P* < 0.05). The N:P in the plant was positively correlated with SOC, TN, SOC:TP, TN:TP, MAT, and MAP and negatively correlated with TP, SOC:TN, and pH (*P* < 0.05) (Fig. 8). In addition, soil SOC, TN, TP and their ratios were influenced by pH, MAT and MAP (Fig. 8).

### Interaction of nutrients between plant tissues

Plant C, N and P concentrations and their ratios were influenced by each plant tissue and showed a synergistic trade-off relationship. Synergistic relationship indicates that two plant tissues promote each other, while trade-off relationship indicates that two plant tissues inhibit each other (Fig. S2).

In the eastern section of the Qilian Mountains, plant leaves had a weak synergistic relationship with stems, branches, fine roots and thick roots in terms of C absorption and with stems and branches in terms of P absorption (*r* < 0, *P* > 0.1). Plant leaves had a strong synergistic relationship with fine roots and thick roots in terms of P absorption (*r* > 0, *P* < 0.05). In addition, plant leaves had a strong trade-off relationship with stems, fine roots and thick roots in terms of N absorption (*r* < 0, *P* < 0.05).

In the middle section of the Qilian Mountains, plant leaves were strongly synergistic with stems and branches in terms of C absorption; with fine roots and thick roots in terms of N absorption; and with stems, branches, fine roots and thick roots in terms of P absorption (*r* > 0, *P* < 0.05). Plant leaves had a weak synergistic effect with thick roots in terms of C absorption and with stems and branches in terms of N absorption (*r* > 0, *P* > 0.1). In addition, plant leaves had a strong trade-off with fine roots in terms of C absorption (*r* < 0, *P* < 0.05).

In the western section of the Qilian Mountains, plant leaves were strongly synergistic with branches and fine roots in terms of C absorption and with stems, branches, fine roots and thick roots in terms of N absorption (*r* < 0, *P* < 0.05). In addition, plant leaves had a strong trade-off with thick roots in terms of C absorption and with stems, branches, fine roots and thick roots in terms of P absorption (*r* < 0, *P* < 0.05).

### Explanation rate of soil and climate on plant tissue stoichiometric characteristics

**Fig. 9:**
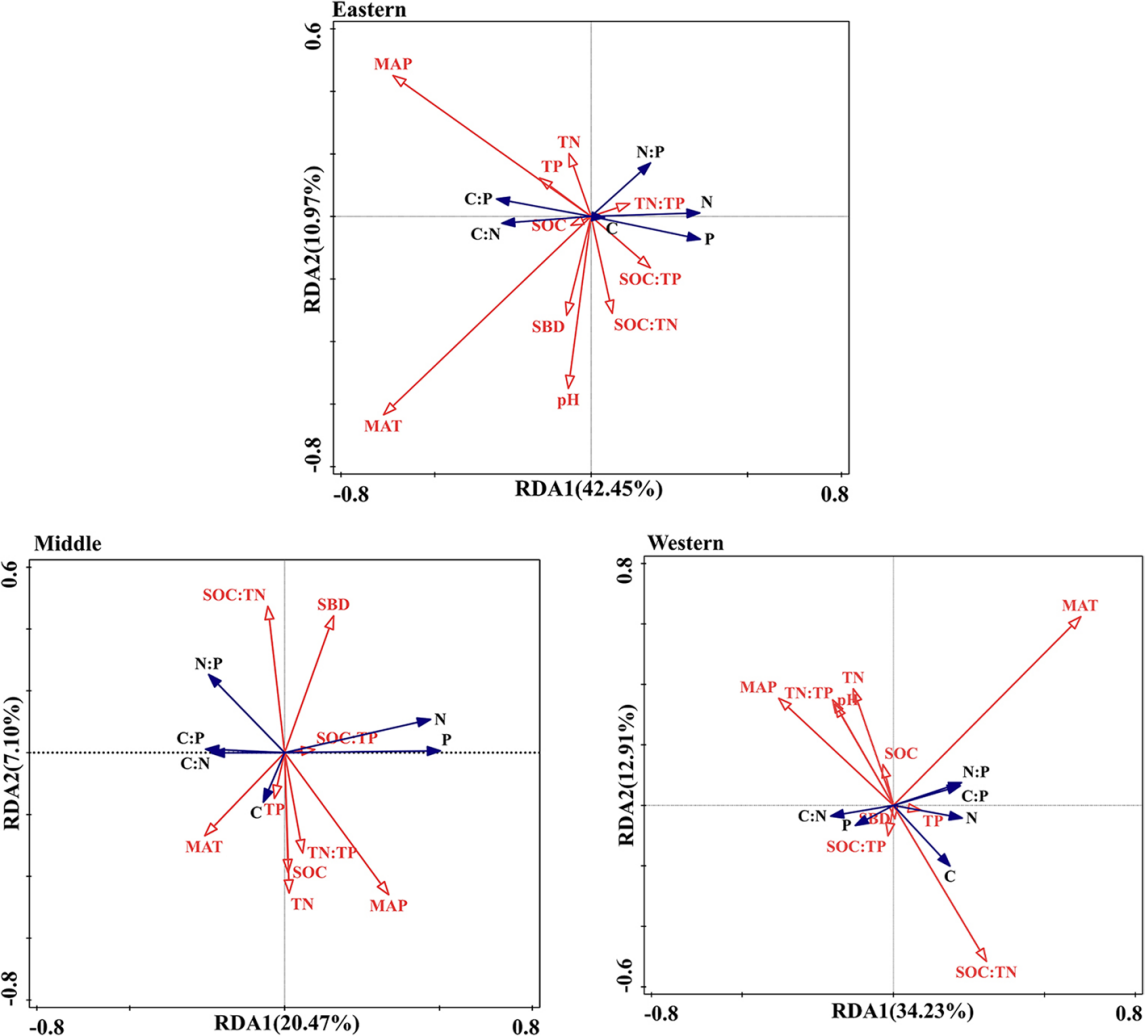
Correlation between plant tissue stoichiometric characteristics and soil, climate in the Qilian Mountains. (Note: The blue line shown the C, N, P contents and its ratio in plants, the red lines represented environmental factors, including soil physical factors and C, N, P contents and its ratio, and climatic factors of mean annual temperature and mean annual precipitation.)

The C, N and P in the plant tissues were influenced by soil and climate. In the eastern section of the Qilian Mountains, the C of the plant leaves, branches, and fine roots was positively correlated with SOC, TN, TP, MAT, and MAP and negatively correlated with pH and SBD. Plant fine root and stem N were negatively correlated with SOC, TN, TP, and MAT. Plant stem and leaf P were positively correlated with SOC, TN, TP, and MAT and negatively correlated with SBD (Table S3). The first principal component axis in the eastern section of the Qilian Mountains explained 42.45% of the total variability. The variability in plant tissue C, N, and P could be explained by MAT, MAP, pH, and SOC. The contribution rates of MAT, MAP, SOC and pH were 42.00%, 35.20%, 26.20% and 13.10%, respectively (Fig. 9).

In the middle section of the Qilian Mountains, the C of the plant leaves, branches, and thick roots was positively correlated with TP and pH and negatively correlated with SOC and TN. Plant leaf N was positively correlated with TP, SBD, and MAP and negatively correlated with SOC and MAT. The P of the plant stems, leaves, and fine roots was positively correlated with TP, pH, SBD, and MAT and negatively correlated with TN and MAP (Table S3). The first principal component axis in the middle Qilian Mountains explained 20.47% of the total variability. The variability in plant tissue C, N, and P could be explained by MAP, TN:TP, and SOC:TP. The contribution rates of MAP, SOC:TN and TN:TP were 29.40%, 23.70% and 12.20%, respectively (Fig. 9).

In the western section of the Qilian Mountains, the C of the plant leaves, branches and fine roots was positively correlated with TP, pH and SBD and negatively correlated with SOC, TN, MAT and MAP. The N of the plant stems, leaves and branches was positively correlated with TP and SBD and negatively correlated with SOC, TN, MAT and MAP. The P in the plant stems and leaves was positively correlated with TP and SBD and negatively correlated with SOC, TN, MAT and MAP. In addition, the P of the plant branches, fine roots and thick roots was positively correlated with SOC and MAP and negatively correlated with TN, TP, pH, SBD and MAT (Table S3). The first principal component axis explained 34.23% of the total variability in the western section of the Qilian Mountains. The variability in plant tissue C, N, and P could be explained by MAT, MAP, and SOC:TN. The contribution rates of MAT, MAP and SOC:TN are 36.30%, 19.00% and 16.20%, respectively (Fig. 9).

## Discussion

### Spatial patterns of soil and plant nutrient characteristics

SOC, TN, and TP in the different soil layers on the eastern margin of the Qinghai-Tibet Plateau showed a decreasing trend with increasing latitude (Table 3, Fig. 4), a result consistent with those of previous studies [39–41]. Soil SOC and TN contents are mainly regulated by organic matter accumulation and microbial activities [42, 43], while MAT and MAP are the main abiotic factors that stimulate organic matter accumulation and microbial activities [26, 44]. SOC also showed a decreasing trend with increasing soil depth because SOC mainly originates from the organic matter produced by the decomposition of soil animals and their manure, microorganisms and plant residues, which are much more abundant in surface soil than in deeper soil. With increasing soil depth, the capacity of organic matter input decreases, and soil organic matter and SOC contents gradually decrease [45, 46]. At latitude, the trend of soil P is eastern > middle > western (Table 3, Fig. 4). Because the temperature in the study area increases and precipitation decreases with latitude, higher phosphorus leaching and weathering rates result [47, 48].

More attention has been given to patterns of plant stoichiometry changes while studying the patterns of soil stoichiometry distribution [49, 50]. In this study, it was found that the C of the plants as well as the plant stems, leaves and branches decreased sharply with increasing latitude (Table 4, Fig. 5). Plants need to increase their structural carbon compounds to overcome soil drought caused by increasing latitude for better growth [51]. In addition, plant N, P, and N:P and leaf N and P contents increased and leaf N:P decreased with latitude (Table 5, Fig. 6), a phenomenon that can be well explained by the temperature-plant physiology hypothesis [4]. Plants usually accumulate more N in their tissues under cold conditions to compensate for the decrease in photosynthetic rate caused by low temperatures [23, 24]. Similar results were obtained at a regional scale, and the leaf N and P contents and N:P ratios showed a single-peaked curve or linear distribution along geographical and climatic gradients [52, 53]. In addition, leaf N:P ratios have been used as indicators of N limitation or P limitation in ecosystems, that is, N:P ratios < 14 indicate N limitation, and N:P ratios > 16 indicate P limitation [54]. In our study, leaf N:P ratios in the Qilian Mountains were below 14, which indicated N-limited plant growth in the Qilian Mountains (Table 5, Fig. 6).

### Characteristics of nutrient distribution patterns among plant tissues

There were differences in nutrient distribution trade-offs among plant tissues in the same region (Fig. 7). The nutrient allocation strategies in plant tissues under different environmental conditions tend to be based on the "optimal partitioning principle". Of that in the different plant tissues, the C concentration in the thick roots in the eastern section of the Qilian Mountains region was the lowest, accounting for only 5.97% (Fig. 7). Because the thick roots of plants are in direct contact with soil, which can provide C elements to plants by soil, plants store more C in leaves for photosynthesis to obtain energy and nutrients for survival [11]. There are more arid areas in the middle and western sections of the region, and the soil provides less C for plants; therefore, plants distribute C equally in all tissues for survival needs. The plant nutrient distribution in the middle section of the Qilian Mountains was similar to that in the eastern section, with C, N and P contents in the leaves at high levels (Fig. 7). The N concentration of the plant stems in the western section accounted for 25.80% of the total plant N, which was higher than that in the middle and eastern sections (Fig. 7). This result occurred because plants in a relatively arid region require more nutrients to maintain normal plant growth. Stems play a role in transporting nutrients during plant growth [14]. A lack of N will lead to reddening of stems and weakened transport capacity, which will affect photosynthesis of plant leaves and respiration of a whole plant [12]. The P concentrations of the thick roots and fine roots in the eastern section of the Qilian Mountains were higher than those in the middle and western sections. Plant roots are closely linked to the soil, and they are supplemented by soil P content. The plants in relatively arid western section consumed more nutrients from the roots to survive [55, 56].

### Coupling relationships between plant stoichiometric characteristics and environmental factors

The results showed that soil and climate dominated the changes in plant tissue stoichiometry characteristics (Fig. 8). Terrestrial plants directly absorb essential elements from the soil, and many scholars believe that soil stoichiometric characteristics determine plant N and P contents [4, 20]. Furthermore, plant C, N, and P are influenced by MAT and MAP. Plants require appropriate temperature and water for respiration, photosynthesis, and transpiration during growth [57]. The plant leaf N content and N:P ratio were negatively correlated with soil TN and TN:TP (*P* < 0.05), showing that soil TN and TN:TP supply the N and P content required for plant growth. Leaves, as the main site for photosynthesis in plants, need more N to synthesize ATP and NADPH to maintain effective photosynthetic capacity. The thick root N was positively correlated with soil TN and N:P (Fig. S2). This phenomenon can be explained by the fact that thick roots are an important channel for direct material exchange between plants and soil, and they play an important role in transferring photosynthetic products and absorbing effective soil nutrients [58, 59].

The interaction of stoichiometric characteristics among plant tissues showed synergistic trade-offs. The leaves and branches, fine roots and thick roots of the plants in the study area absorbed C in a synergistic relationship (Fig. S2). This phenomenon is better explained by the photosynthesis of CO_2_ and H_2_O in plant leaves to produce carbohydrates, which are supplied to various plant tissues through plant stems [60]. The plant leaves had a trade-off with stems, fine roots, and thick roots in terms of N absorption. Because N and P are mostly absorbed and used by plants from the soil and the tissues compete with each other [61], they were in a trade-off relationship. Overall, plant tissue nutrient content was strongly influenced by soil C, N, and P content as well as soil pH and SBD. At the same time, MAT had a positive effect on plant tissues, and MAP had more negative than positive effects (Table S3, Fig. 8, Fig. S2). Studying the stoichiometric characteristics of plant tissues allows us to understand how plants regulate nutrient partitioning strategies to adapt to their surroundings from new perspectives.

The results of the RDA suggested the main controlling factors influencing the changes in plant stoichiometric characteristics in the three regions (Table S3, Fig. 9). The stoichiometric characteristics of the plants in the eastern and western sections of the Qilian Mountains were mainly influenced by temperature and precipitation. The interpretation rates of MAT to the eastern and western sections were 42.00% and 36.30%, and the interpretation rates of MAP to to the eastern and western sections were 35.20% and 19.00%. Previous dendrochronological and tree-ring climatology studies have shown that the growth of Qinghai spruce is highly sensitive to precipitation [62, 63]. In the western section of the Qilian Mountains, temperatures are high, and precipitation is low, resulting in plants being more restricted by water (Fig. 1, Fig. S1). Therefore, the water availability of Qinghai spruce affects the growth of plants in this region. Plant stems and leaf P in the three regions were positively correlated with TP and MAT (Fig. 9) and negatively correlated with MAP. This result indicates that the P morphology of plant stems and leaves was essentially the same [4, 64]. Plant tissue P content is directly related to soil nutrient supply (Table S3, Fig. 9), while temperature affects soil nutrients, the accumulation of organic matter and microbial activity [22]. Soil N and P leaching increased with increasing precipitation (Table 3, Fig. 4), resulting in a decrease in soil N and P concentrations [65]. This result may have been responsible for the negative correlation between stem and leaf P and MAP. In addition, branch N and P were negatively correlated with soil TN and TP because root uptake is the main source of N and P from soil to plant. Plant tissues are affected by regional MAT and MAP, and in arid regions, precipitation closely affects soil nutrient content, which in turn acts on plant tissue nutrient content [55]. Therefore, precipitation plays a key role in regulating nutrient flow in plant tissues in arid regions. Therefore, studying the nutrient content of plant tissues and the characteristics of the coupling relationship with environmental factors can provide a better understand the driving forces behind plant resource allocation and an effective basis for future plant nutrient control.

## Conclusion

The investigation of soil and plant C:N:P stoichiometry was conducted under three environmental conditions in the Qilian Mountains on the eastern margin of the Qinghai-Tibet Plateau. The plant and soil stoichiometric characteristics showed spatial trends (*P* < 0.05), and plant and soil stoichiometric characteristics were consistent latitudinally. The results of the pattern of variation in plant stoichiometric characteristics along a latitudinal gradient supported the temperature-plant physiology hypothesis. In addition, plant stoichiometric characteristics are influenced by the role of environmental factors such as soil and climate, and individual plant tissues interact with each other and have synergistic trade-off relationships. The nutrient allocation strategies in plant tissues under different environmental conditions tend to be based on the "optimal partitioning principle". The ecological patterns of plant-soil C:N:P stoichiometry in different regions can help us better understand the nutrient allocation patterns in arid and semiarid forest ecosystems from a wide geographic scale.

## Supplementary Information

**Additional file 1.** 

**Additional file 2.**

## Data Availability

The datasets used and/or analyzed during the current study available from the corresponding author on reasonable request.
