# Supplementary material for: Spatial characteristics of nutrient allocation for Picea crassifolia in soil and plants on the eastern margin of the Qinghai-Tibet Plateau
Source: BMC Plant Biol. 2023 Apr 17;23:199. doi: 10.1186/s12870-023-04214-x (PMC10108462; doi:10.1186/s12870-023-04214-x)
Supplement: Supplementary file 1 — Additional file 1. [file 12870_2023_4214_MOESM1_ESM.zip › Supplementary table/Table S1.docx]

**Table S1**

The information of weather observation site

| Site name | Latitude(°) | Longitude(°) | Distance(km) |
| --- | --- | --- | --- |
| Wushaoling Station (eastern section) | 37.20 | 102.87 | 36.69 |
| Yeniugou Station (middle section) | 38.41 | 99.58 | 37.06 |
| Jiuquan Station (western section) | 39.76 | 98.48 | 41.16 |

Note: Distance represents the distance from the meteorological station to the sampling point
