# Supplementary material for: Spatial characteristics of nutrient allocation for Picea crassifolia in soil and plants on the eastern margin of the Qinghai-Tibet Plateau
Source: BMC Plant Biol. 2023 Apr 17;23:199. doi: 10.1186/s12870-023-04214-x (PMC10108462; doi:10.1186/s12870-023-04214-x)
Supplement: Supplementary file 1 — Additional file 1. [file 12870_2023_4214_MOESM1_ESM.zip › Supplementary table/Table S2.docx]

**Table S2**

Synergistic trade-offs among plant tissues (r was the correlation coefficient, P was significance)

| relationship | | r | p |
| --- | --- | --- | --- |
| synergy | strong synergy | >0 | P<0.05 |
|  | medium synergy |  | 0.05<P<0.1 |
|  | weak synergy |  | 0.1<P |
| trade-off | strong trade-off | <0 | P<0.05 |
|  | medium trade-off |  | 0.05<P<0.1 |
|  | weak trade-off |  | 0.1<P |

Note: When the correlation coefficient r>0, it is considered to be a synergistic relationship; when P<0.05, it is considered to be a strong synergistic relationship. When 0.05<P<0.1, it is considered that there is a moderate synergistic relationship between them. When 0.1<P, they are considered to be weakly synergistic. When the correlation coefficient r<0, it is considered to be a trade-off relationship. When P<0.05, it is considered to be a strong trade-off relationship. When 0.05<P<0.1, it is considered that there is a medium trade-off. When 0.1<P, the relationship between them is considered to be weak.
