# Supplementary material for: Spatial characteristics of nutrient allocation for Picea crassifolia in soil and plants on the eastern margin of the Qinghai-Tibet Plateau
Source: BMC Plant Biol. 2023 Apr 17;23:199. doi: 10.1186/s12870-023-04214-x (PMC10108462; doi:10.1186/s12870-023-04214-x)
Supplement: Supplementary file 1 — Additional file 1. [file 12870_2023_4214_MOESM1_ESM.zip › Supplementary table/Table S3.docx]

**Table S3**

Correlation coefficients of plant tissue stoichiometric characteristics (C, N, P and C:N:P) with soil characteristics, mean annual temperature (MAT,°C) and mean annual precipitation (MAP, mm) in the Qilian Mountains ( * Correlation is significant at the 0.05 level, ** Correlation is significant at the 0.01 level )

| Eastern section | | SOC | TN | TP | SOC:TN | SOC:TP | TN:TP | pH | SBD | MAT | MAP |
| --- | --- | --- | --- | --- | --- | --- | --- | --- | --- | --- | --- |
| C | Stems | 0.95** | 0.67** | 0.21 | -0.25 | 0.39** | 0.61** | 0.54** | -0.12 | 0.23 | -0.64** |
|  | Leaf | .062** | 0.97** | 0.84** | -0.95** | -0.48** | -0.03 | -0.17 | -.92** | 0.68** | 0.32* |
|  | Branch | 0.57** | 0.57** | 0.84** | -0.33* | -0.64** | -0.55** | 0.52** | -0.54** | 0.96** | -0.11 |
|  | Fine root | 0.47** | 0.91** | 0.78** | -0.99** | -0.49** | -0.03 | -0.36** | -0.94** | 0.57** | 0.48** |
|  | Thick root | -0.29* | 0.33* | 0.37** | -0.75** | -0.51** | -0.18 | -0.91** | -0.71** | 0.09 | 0.93** |
| N | Stems | -0.95** | -0.63** | -0.43** | 0.16 | -0.09 | -0.21 | -0.78** | 0.16 | -0.55** | 0.67** |
|  | Leaf | -0.05 | 0.46** | 0.84** | -0.71** | -0.96** | -0.74** | -0.39** | -0.87** | 0.74** | 0.75** |
|  | Branch | 0.64** | 0.13 | -0.35* | 0.28* | 0.81** | 0.80** | 0.62** | 0.45** | -0.26 | -0.88** |
|  | Fine root | -0.80** | -0.96** | -0.88** | 0.78** | 0.45** | 0.09 | -0.18 | 0.81** | -0.83** | -0.03 |
|  | Thick root | 0.95** | 0.61** | 0.18 | -0.16 | 0.41** | 0.58** | 0.62** | -0.04 | 0.23 | -0.71** |
| P | Stems | 0.68** | 0.80** | 0.94** | -0.62** | -0.64** | -0.39** | 0.33* | -0.75** | 0.97** | 0.02 |
|  | Leaf | 0.60** | 0.89** | 0.98** | -0.83** | -0.69** | -0.35* | 0.02 | -0.91** | 0.91** | 0.27 |
|  | Branch | -0.38** | 0.07 | -0.14 | -0.47** | 0.02 | 0.28 | -0.94** | -0.29* | -0.42** | 0.71** |
|  | Fine root | -0.48** | 0.08 | 0.06 | -0.55** | -0.27 | -0.02 | -0.99** | -0.46** | -0.21 | 0.89** |
|  | Thick root | 0.85** | 0.43** | -0.05 | 0.01 | 0.61** | 0.72** | 0.62** | 0.16 | 0.01 | -0.80** |
|  |  |  |  |  |  |  |  |  |  |  |  |
|  |  |  |  |  |  |  |  |  |  |  |  |
| Middle section | | SOC | TN | TP | SOC:TN | SOC:TP | TN:TP | pH | SBD | MAT | MAP |
| C | Stems | -0.20 | -0.22 | 0.30* | 0.14 | -0.43** | -0.38** | 0.61** | -0.14 | 0.62** | -0.65** |
|  | Leaf | -0.50** | -0.85** | 0.94** | 0.76** | -0.98** | -0.96** | 0.74** | -0.28 | 0.42** | -0.64** |
|  | Branch | -0.69** | -0.29* | 0.35* | 0.16 | -0.14 | -0.09 | -0.02 | 0.42** | -0.50** | 0.07 |
|  | Fine root | 0.66** | 0.68** | -0.42** | -0.71** | 0.52** | 0.55** | -0.94** | 0.71** | -0.85** | 0.97** |
|  | Thick root | -0.63** | -0.52** | 0.79** | 0.29* | -0.66** | -0.57** | 0.26 | 0.48** | -0.27 | -0.16 |
| N | Stems | -0.65** | -0.49** | 0.30* | 0.50** | -0.18 | -0.21 | 0.24 | -0.17 | -0.07 | -0.19 |
|  | Leaf | -0.31* | 0.08 | 0.29* | -0.32* | -0.11 | -0.01 | -0.26 | 0.90** | -0.66** | 0.30* |
|  | Branch | -0.04 | 0.18 | -0.08 | -0.27 | -0.01 | 0.04 | 0.31* | 0.07 | 0.42** | -0.40** |
|  | Fine root | -0.05 | -0.40** | 0.70** | 0.25 | -0.70** | -0.65** | 0.20 | 0.26 | -0.05 | -0.09 |
|  | Thick root | -0.34* | 0.47** | -0.43** | -0.58** | 0.53** | 0.61** | -0.17 | 0.50** | -0.25 | 0.05 |
| P | Stems | -0.05 | -0.57** | 0.39** | 0.69** | -0.38** | -0.47** | 0.14 | -0.49** | 0.05 | -0.02 |
|  | Leaf | -0.17 | -0.51** | 0.30* | 0.60** | -0.50** | -0.54** | 0.84** | -0.81** | 0.98** | -0.86** |
|  | Branch | 0.34* | -0.09 | 0.03 | 0.18 | -0.25 | -0.28* | 0.43** | -0.54** | 0.74** | -0.46** |
|  | Fine root | -0.14 | -0.80** | 0.67** | 0.88** | -0.80** | -0.86** | 0.72** | -0.74** | 0.69** | -0.63** |
|  | Thick root | -0.02 | -0.24 | 0.19 | 0.26 | -0.38** | -0.38** | 0.66** | -0.48** | 0.83** | -0.70** |

| Western section | | SOC | TN | TP | SOC:TN | SOC:TP | TN:TP | pH | SBD | MAT | MAP |
| --- | --- | --- | --- | --- | --- | --- | --- | --- | --- | --- | --- |
| C | Stems | 0.26 | -0.06 | -0.51** | 0.09 | 0.33* | 0.02 | -0.10 | -0.60** | 0.32* | -0.30* |
|  | Leaf | -0.54** | -0.98** | 0.51** | 0.91** | -0.33* | -0.89** | -0.22 | -0.02 | -0.86** | -0.13 |
|  | Branch | -0.98** | -0.48** | 0.90** | 0.25 | -0.93** | -0.90** | 0.62** | 0.68** | -0.82** | -0.77** |
|  | Fine root | -0.86** | -0.77** | 0.78** | 0.60** | -0.72** | -0.99** | 0.28* | 0.38** | -0.91** | -0.57** |
|  | Thick root | 0.67** | 0.94** | -0.64** | -0.83** | 0.48** | 0.96** | 0.05 | -0.13 | 0.92** | 0.27 |
| N | Stems | -0.88** | -0.78** | 0.87** | 0.60** | -0.76** | -0.98** | 0.25 | 0.48** | -0.97** | -0.45** |
|  | Leaf | -0.95** | -0.66** | 0.90** | 0.46** | -0.85** | -0.97** | 0.41** | 0.58** | -0.93** | -0.60** |
|  | Branch | -0.53** | -0.58** | 0.72** | 0.52** | -0.45** | -0.57** | -0.09 | 0.45** | -0.81** | 0.19 |
|  | Fine root | -0.25 | -0.97** | 0.21 | 0.96** | -0.02 | -0.72** | -0.47** | -0.34* | -0.66** | 0.04 |
|  | Thick root | -0.78** | 0.21 | 0.79** | -0.42** | -0.90** | -0.34* | 0.89** | 0.98** | -0.38** | -0.66** |
| P | Stems | -0.30* | -0.39** | 0.54** | 0.38** | -0.25 | -0.30* | -0.21 | 0.37** | -0.59** | 0.42** |
|  | Leaf | -0.87** | -0.11 | 0.94** | -0.08 | -0.92** | -0.56** | 0.67** | 0.96** | -0.67** | -0.48** |
|  | Branch | 0.53** | -0.52** | -.42** | 0.71** | 0.69** | 0.06 | -0.98** | -0.76** | -0.04 | 0.82** |
|  | Fine root | 0.29* | -0.72** | -0.18 | 0.86** | 0.48** | -0.19 | -0.91** | -0.62** | -0.29* | 0.70** |
|  | Thick root | 0.79** | -0.18 | -0.81** | 0.40** | 0.91** | 0.36** | -0.88** | -0.99** | 0.40** | 0.64** |
