# Supplementary material for: Spatial characteristics of nutrient allocation for Picea crassifolia in soil and plants on the eastern margin of the Qinghai-Tibet Plateau
Source: BMC Plant Biol. 2023 Apr 17;23:199. doi: 10.1186/s12870-023-04214-x (PMC10108462; doi:10.1186/s12870-023-04214-x)
Supplement: Supplementary file 2 — Additional file 2. [file 12870_2023_4214_MOESM2_ESM.zip › Supplementary figure/Fig. S1.docx]

**Fig. S1** Interannual variation of mean annual temperature (MAT, ℃) and mean annual precipitation (MAP, mm) in the Qilian Mountains
