# Supplementary material for: Spatial characteristics of nutrient allocation for Picea crassifolia in soil and plants on the eastern margin of the Qinghai-Tibet Plateau
Source: BMC Plant Biol. 2023 Apr 17;23:199. doi: 10.1186/s12870-023-04214-x (PMC10108462; doi:10.1186/s12870-023-04214-x)
Supplement: Supplementary file 2 — Additional file 2. [file 12870_2023_4214_MOESM2_ESM.zip › Supplementary figure/Fig. S2.docx]

**Fig. S2** Interaction of tissue stoichiometry characteristics in relation in the Qilian Mountains

(Note: S = stem, L = leaf, B = branch, FR = fine roots, TR = thick roots, SC = stem carbon concentration, LC = leaf carbon concentration, BC = branch carbon concentration, FRC = fine roots carbon concentration, TRC = thick roots carbon concentration, SN = stem nitrogen concentration, LN = leaf nitrogen concentration, BN = branch nitrogen concentration, FRN = fine roots nitrogen concentration, TRN = thick roots nitrogen concentration, SP = stem phosphorus concentration, LP = leaf phosphorus concentration, BP = branch phosphorus concentration, FRP = fine roots phosphorus concentration, TRP = thick roots phosphorus concentration)
